# TGFBI functions similar to periostin but is uniquely dispensable during cardiac injury

**DOI:** 10.1371/journal.pone.0181945

**Published:** 2017-07-27

**Authors:** Jennifer A. Schwanekamp, Angela Lorts, Michelle A. Sargent, Allen J. York, Kelly M. Grimes, Demetria M. Fischesser, Jason J. Gokey, Jeffrey A. Whitsett, Simon J. Conway, Jeffery D. Molkentin

**Affiliations:** 1 From the Department of Pediatrics, Cincinnati Children’s Hospital Medical Center, University of Cincinnati, Cincinnati, Ohio, United States of America; 2 Herman B. Wells Center for Pediatric Research, Indiana University School of Medicine, Indianapolis, Indiana, United States of America; 3 Howard Hughes Medical Institute, Cincinnati Children’s Hospital Medical Center, Cincinnati, Ohio, United States of America; Albert Einstein College of Medicine, UNITED STATES

## Abstract

Extracellular matrix production and accumulation stabilize the heart under normal conditions as well as form a protective scar after myocardial infarction injury, although excessive extracellular matrix accumulation with long-standing heart disease is pathological. In the current study we investigate the role of the matricellular protein, transforming growth factor beta-induced (TGFBI), which is induced in various forms of heart disease. Additionally, we sought to understand whether TGFBI is functionally redundant to its closely related family member periostin, which is also induced in the diseased heart. Surgical models of myocardial infarction and cardiac pressure overload were used in mice with genetic loss of *Postn* and/or *Tgfbi* to examine the roles of these genes during the fibrotic response. Additionally, cardiac-specific TGFBI transgenic mice were generated and analyzed. We observed that deletion of *Tgfbi* did not alter cardiac disease after myocardial infarction in contrast to greater ventricular wall rupture in *Postn* gene-deleted mice. Moreover, *Tgfbi* and *Postn* double gene-deleted mice showed a similar post-myocardial infarction disease phenotype as *Postn*-deleted mice. Over-expression of TGFBI in the hearts of mice had a similar effect as previously shown in mice with periostin over-expression. Thus, TGFBI and periostin act similarly in the heart in affecting fibrosis and disease responsiveness, although TGFBI is not seemingly necessary in the heart after myocardial infarction injury and is fully compensated by the more prominently expressed effector periostin.

## Introduction

Cardiovascular disease remains the leading cause of death in the United States affecting more than 85 million people at an estimated annual cost of more than $300 billion [1]. A wide array of cardiovascular disease etiologies often culminate in heart failure, which is characterized by progressive cardiomyocyte cell death and the accumulation of excessive extracellular matrix (ECM) with adverse ventricular remodeling [2, 3]. However, ECM deposition after acute myocardial infarction (MI) injury is critical in facilitating scar formation to protect the ventricular wall from rupture. In longstanding chronic heart disease states, excess deposition of ECM leads to decreased ventricular wall compliance with reduced function [2]. Current therapeutics that specifically target excessive fibrosis and ECM production in chronic heart failure are lacking but desperately needed. Hence, it remains critical to understand the underlying mechanisms and associated genes whereby ECM accumulates in the heart during chronic disease states.

Transforming growth factor beta-induced (TGFBI) is a 68 kDa matricellular protein and a member of the fasciclin domain containing protein family that also includes periostin [4]. As a secreted protein that resides within the ECM, TGFBI affects cell migration and proliferation in models of cancer [5–9]. TGFBI shares substantial amino acid sequence similarity with periostin and a highly conserved protein domain structure [10]. Both TGFBI and periostin are evolutionarily conserved with a single paralog being present in *Drosophila*, suggesting that such proteins play an important role in biology [11]. Several studies have demonstrated the importance of periostin in facilitating collagen maturation and matrix production with proper scar formation in the hearts of mice after MI injury [12, 13]. In addition to facilitating collagen organization and cross-linking, periostin can interact with fibronectin and tenascin-C in generating additional ECM structural capacity [14]. Characterization of TGFBI in disease has primarily focused on corneal dystrophy where human mutations in the gene encoding this protein cause aberrant folding leading to TGFBI aggregation in the cornea resulting in blindness [15–17]. *Tgfbi* null mice showed morphologically altered lungs from prior aberrant developmental maturation as well as augmented myofibroblast numbers throughout this organ [18].

Given the significant similarity between periostin and TGFBI, we hypothesized that these proteins may have overlapping functions during cardiac ventricular remodeling. Previous studies have shown that periostin is robustly increased after injury in the heart and is required for adequate scar formation after MI [12, 13, 19, 20]. TGFBI mRNA is also induced in the injured heart [21], although, there have been no functional studies specifically addressing the role of this protein in the heart.

## Materials and methods

### Mice

Mice with genetic deletion of *Postn* and *Tgfbi* were previously described and were maintained as a mixed C57Bl/6-Sv129 background [12, 18]. To generate transgenic mice over-expressing TGFBI, the human TGFBI cDNA was cloned downstream of a modified tetracycline inducible α-myosin heavy chain (MHC) promoter construct, which was injected into newly fertilized mouse oocytes in the FVB/N background [22]. This allowed for the tetracycline/doxycycline inducible expression of TGFBI specifically in the heart when crossed with transgenic mice expressing the tet-transactivator (tTA) protein, also driven by the α-MHC promoter [22]. Primers used for genotyping were 5’-AAGTCTCTCCAAGGTGACAAGC-3’ and 5’-AGAAGGACACCTAGTCAGAC-3’ for the TGFBI transgene and 5’-AGCGCATTAGAGCTGCTTAATGAGGTC-3’ and 5’-GTCGTAATAATGGCGGCATACTATC-3’ for the tet-transactivator transgene.

### Animal husbandry and ethics statement

Mice were housed in standard barrier rack cages supplied with Purina Rodent Chow 5001 with automatic watering dispensers. Cages were observed daily and were changed weekly by certified veterinary technicians at Cincinnati Children’s Hospital Medical Center. Mice were also closely assessed for their well-being monitored by adequate physical activity and food intake on a daily basis. Housing conditions and husbandry conformed to AAALAC standards and the institution's ongoing certification by this organization, as well as by the standard guidelines from the Office of Laboratory Animal Welfare (http://grants.nih.gov/grants/olaw/animal_use.htm). All animal experimentation, including this specific study, was approved by the Office of Research Compliance and Regulatory Affairs and by the Cincinnati Children’s Hospital Institutional Animal Care and Use Committee (Protocol Number: IACUC 2016–0069, expires 11–2019). No human subjects were used.

### Echocardiography and surgical models of MI and TAC

Echocardiographic measurements were performed under 2.0% isoflurane anesthesia using a Hewlett Packard SONOS 5500 instrument [12]. Transverse aortic constriction (TAC) surgery as a model of cardiac pressure overload was performed in 8–12 week-old mice and cardiac functional characteristics were monitored with M-mode echocardiography every 2 weeks until the experiment was completed 12 weeks later [12, 23]. The TAC procedure was performed under 2% isoflurane-induced anesthesia. TAC operated mice were monitored daily by veterinary technicians for signs of distress and heart failure including lethargy, difficulty breathing and decreased physical activity. When any of these metrics differed substantially between sham and TAC operated animals, treatments including analgesic, IV fluids and soft diet were administered. Mice were under the care of a veterinarian and were monitored for improvement. If there was no improvement in symptoms after 24 hours, the mice were sacrificed. For MI surgery, the left coronary artery of 6–8 week-old mice was permanently occluded and M-mode echocardiography was used to monitor cardiac functional characteristic at 1, 3, 5 and 8 weeks post-surgery [12, 23]. For the MI procedure, 2% isoflurane-induced anesthesia was given to the mice until sedated as assessed by toe pinch. At the end of all surgical procedures, mice were given the pain medicine buprenex at a final concentration of 0.03 mg/ml by veterinary technicians. Mice were then transferred to a 30°C chamber and closely monitored to allow overnight recovery. The next day, mice were transferred back to standard housing and again monitored daily by veterinary technicians. Equal ratios of male and female mice were used in all experiments and procedures. At the end of the 8 week experiment, mice were anesthetized with 2.0% isoflurane until fully sedated before sacrifice. The mice were then cervically dislocated and the hearts were injected with 1 M KCl to arrest them in diastole. Hearts were either fixed in 10% buffered formalin for histological analysis or flash frozen for protein analysis.

### Survival study

Mice were assessed daily by AAALAC certified veterinary technicians at Cincinnati Children’s Hospital Medical Center for health and well-being including physical activity and food and water intake. Complications associated with surgical intervention were also monitored including lethargy and signs of heart failure. Mice were under veterinarian care to manage any signs of distress and provided with analgesics as needed or IV fluids with a special soft diet to accommodate recovery from surgical procedures. CCHMC’s IACUC committee has reviewed and approved the experiments for this study including anticipated mortality rates due to both surgical complications and study related events. Survival was monitored for 1 week after MI surgery in which mice died abruptly from ventricular wall rupture. To produce statistically meaningful numbers of mice that survived the 8 week experiment, a total of 147 mice were used for MI surgery (WT = 56 (26), *Tgfbi*^*-/-*^ = 45 (24), *Postn*^*-/-*^ = 23 (17) and DKO = 23 (19)) with the number of mice that died included in parentheses. All mice that died were necropsied to assess cardiac ventricular wall rupture as a cause of death. Mouse experimentation to assess cardiac rupture was required due to the lack of *in vitro* models of this process.

### Model of acute lung injury

Lung injury in mice was induced by intranasal administration of 0.05 U of bleomycin (Teva Pharmaceuticals, NDC# 00703-3154-01), a well-known inducer of idiopathic pulmonary fibrosis [24–26], once a week for three weeks under 2.0% isoflurane anesthesia. Lungs were harvested one week after the final treatment and flash frozen before processing for protein analysis. No pain relief was deemed necessary for this procedure given the need to induce a fibrotic response in the lungs and because the procedure does not elicit a noticeable pain response in the mice.

### Cell isolation and Western blotting

Adult cardiomyocytes were isolated from 8-week old wildtype (WT) mice as previously described [27, 28]. Briefly, beating hearts were removed from anesthetized mice (as described above) and cannulated for perfusion with modified Tyrode solution (120 mL NaCl, 5.4 mM KCl, 1.2 mM NaH_2_PO_4_, 5.6 mM glucose, 20 mM NaHCO_3_, 1.6 mM MgCl_2_, 10 mM 2,3-butanedione monoxime (BDM) and 5 mM taurine; buffer A). Hearts were then perfused with buffer A containing liberase blendzyme (Roche, cat# 05401151001) at 37°C. After perfusion, atria were excised and hearts were dissociated into individual cardiomyocytes, filtered and allowed to settle by gravity. The supernatant was then removed and the cardiomyocytes were re-suspended in a modified RIPA buffer (10 mM Tris-HCl, pH 7.5, 150 mM NaCl, 4% glycerol, 0.5 mM sodium metabisulfite, 1% triton X-100, 0.1% sodium deoxycholate, and 0.05% SDS) containing 1x phosphatase inhibitor cocktail 1 and 2 (Millipore, cat# 524624 and 524625), 1 mM DTT and 1x protease inhibitors (Fisher Scientific, cat# 88666) and sonicated (Bioruptor UCD-200) [29].

Adult cardiac fibroblasts were isolated according to protocol 2 of Pinto et al. [30]. Briefly, hearts were isolated from 8-week old WT mice and minced into 12 to 14 pieces in 1x Hanks Balanced Salt Solution (HBSS, HyClone, cat# SH30588.01). The heart pieces were transferred to a digestion solution (1x HBSS, 2 mg/mL collagenase type IV (Worthington, Cat# LS004188), 1.2 U/mL dispase II (Roche, Cat# 10165859001), and 0.9 mM CaCl_2_) and incubated at 37°C for 15 min with gentle rocking. The digestion mix was triturated 12 times using a 10 mL serological pipette and placed back in the incubator. The trituration was repeated two more times for a total digestion period of 45 min and the final trituration was performed with a p1000 pipetteman (Gilson, p1000) [30]. The digestion was transferred to a 15 mL conical tube with 5 mL of culture media containing DMEM (Thermoscientific, cat# SH30284.02) supplemented with 10% FBS (Sigma, Cat# F2442), and 1% Pen/Strep (Fisher Scientific Cat# 30-002Cl). The cells were pelleted at 1000 x g for 5 minutes at room temperature. Following aspiration of the supernatant, the pellet was re-suspended in 10 mL of culture medium and plated on a 10 cm tissue culture dish coated with 0.1% gelatin. Cells were harvested at passage one, scraped with modified RIPA buffer (see above), and sonicated (Bioruptor UCD-200) for 5 minutes in an ice water bath.

Mouse hearts were removed 1 day, 1 week or 2 weeks after MI injury and the area of infarction was dissected to generate protein extracts. The tissue was homogenized in modified RIPA buffer, sonicated for 5 min, centrifuged for 10 min at 12,000 x g at 4°C and the supernatant was used for Western analysis. Protein concentrations were quantified using Bradford reagent (Bio-Rad, Cat#500–0006) according to manufacturer's instructions and then run on a 7.5% SDS-PAGE gel for Western blotting. Primary antibodies for periostin (Novus, 1:1000, Cat#NBP1-30042), TGFBI (Protein Tech (Cat #10188-1-AP or Abcam (Cat# AB170874), 1:1000), sarcomeric α-actin (Abcam (Cat# AB62298), 1:1000), and GAPDH (Fitzgerald (Cat# 10R-G109A), 1:5000) were used for protein detection. Secondary antibodies (LI-COR biosciences (Cat# 926–32211), 1:10,000) were incubated for 1 hour at room temperature prior to imaging on an Odyssey CLx LI-COR scanner [31]. Densitometry was performed for Western blot quantification using Image Studio software version 3.1.

### RNA and QPCR

RNA was isolated from MI- or sham-operated hearts 1 week after surgery and either flash-frozen or stored in RNAlater (Ambion, Cat# AM7021) as previously described [31]. Briefly, hearts were weighed and 1 mL of Trizol (Invitrogen, Cat#15596–026) was added per 50 mg of tissue. Hearts were homogenized and RNA was isolated according to manufacturer's instructions (Invitrogen, Cat#15596–026). The RNA was then run through an RNeasy column (Qiagen, Cat# 74104) including the optional column used with DNase digestion. Four micrograms of RNA were used as a template for cDNA synthesis using the Superscript III first strand synthesis kit (Invitrogen, Cat# 18080–051) according to manufacturer's instructions. Quantitative real time PCR (QPCR) was performed with 2 μL of this first strand cDNA mix diluted 1 to 10 using the SsoAdvanced Universal SYBR Green Supermix (Bio-Rad, Cat# 172–5275). QPCR was run on a Bio-Rad CFX96 C1000 real time Thermo Cycler. Primers used for TGFBI are forward 5’-AGATCTGGCAGTCATAGCTTGGCA-3’ and reverse 5’- TGTGAGCTCCAAGATGCCGTAGTT-3’. Primers for periostin are forward 5’–CAAAGCACACAGTTACCTTTCCAGGG– 3’ and reverse 5’–GCAGGAAACCCACATTGCATGAGA– 3’. Primers used for 18s rRNA (control) are forward 5’- GTAACCCGTTGAACCCCATT-3’ and reverse 5’- CCATCCAATCGGTAGTGACG -3’.

### Immunohistochemistry and histology

Histological and immunohistochemical analyses were performed on formalin fixed 5–6 μm thick paraffin sections, which were then stained with Masson’s trichrome and imaged at 100x or 200x magnification. Fibrosis was quantified from Masson’s trichrome-stained sections using Image J software and graphed as percent fibrotic area [29].

Immunohistochemistry was performed as described previously and the following primary antibodies were used: periostin (Abcam (Cat# AB92460), 1:100), TGFBI (Abcam, 1:100), and α-actinin (Sigma (Cat# A7811), 1:400) [29]. Alexa Fluor secondary antibodies were used at a concentration of 1:200 through 1:400 (Cat# A11031, and A11008). Pictures were taken using confocal microscopy (Nikon A1 Confocal). Cardiomyocyte cross-sectional area was measured on 200x images stained with wheat germ agglutinin-Alexa Fluor 647 Conjugate (Thermo Fisher Scientific (Cat# W32466), 1:200), laminin (Sigma (Cat# L9393), 1:50) and 4,6-diamidino-2-phenylindole, dihydrochloride (DAPI, Invitrogen (D1306), 1:5000). Cardiomyocytes with a visible central nucleus were used to measure cross sectional area using Image J software. Centrally nucleated cardiomyocytes were measured across three fields of each heart.

### Statistical analysis

All results are represented as mean ± SEM. Unpaired Students t-test was performed to compare means between two groups. Analysis of variance (ANOVA) with a Newman-Keuls post-hoc test was used to compare means between multiple groups of the four gene-deleted mouse lines used with surgical intervention, The log rank Mantel-Cox test was used to compare survival curves between groups of mice after surgical intervention. A p-value of less than 0.05 was considered statistically significant.

## Results

### TGFBI expression is increased in the heart after injury

**Fig 1:**
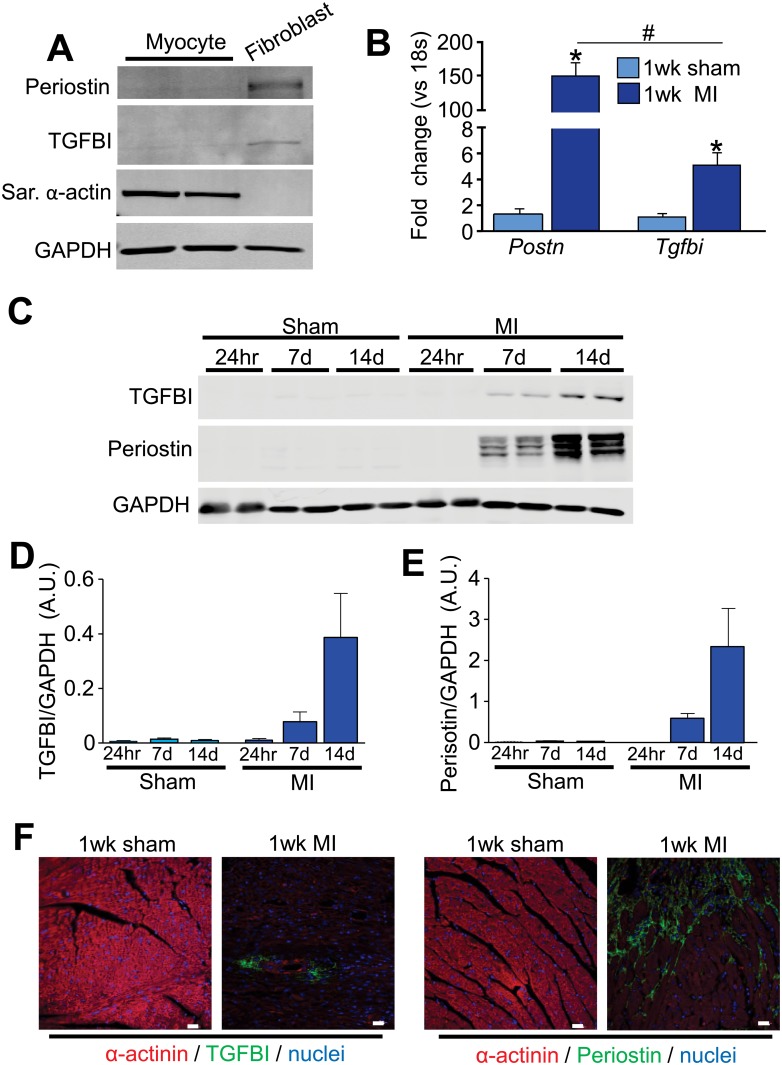
TGFBI and periostin are induced in the heart after injury. (A) Western blot analysis of periostin and TGFBI in isolated adult cardiomyocytes and fibroblasts. Sarcomeric α-actin was used as a control for cardiomyocyte purity and GAPDH was used as a loading control. (B) Quantitative real time PCR for *Postn* and *Tgfbi* from 1 week sham or MI-operated hearts. mRNA levels were normalized to 18s ribosomal RNA. *p<0.05 for both genes in MI-operated animals compared to sham animals using an unpaired Student’s T-test. n = 3 animals. (C) Western blot analysis for periostin and TGFBI in the infarcted areas isolated from hearts 24 hours, 7 and 14 days after MI surgery. GAPDH was used as a loading control. Each lane corresponds to protein from one mouse. (D and E) Quantification of TGFBI (D) and periostin (E) protein levels from the conditions shown in panel C except that 3 hearts were analyzed in total each. (F) Immunohistochemistry for the indicated markers/proteins on sham or MI-operated hearts after 1 week. Images were taken at 400x magnification. Scale = 20 μm.

Periostin expression is induced in a wide array of tissues following injury, and this induction of expression appears to be specific to the tissue resident, activated fibroblast [12, 19, 20]. The closely related family member TGFBI is also induced in areas of injury or cancers of the colon [9], ovary [6], lung [8], breast [7] and within the injured heart [20]. Here we show that similar to periostin [12], TGFBI induction is largely specific to the fibroblast, but not the cardiomyocyte, as assessed by Western blotting of protein extracts from isolated cells from this organ (Fig 1A). *Tgfbi* mRNA levels were also increased in the heart 7 days post-MI injury compared to sham-operated mice, although Postn mRNA levels were induced at substantially higher levels (Fig 1B). The infarct areas of the mouse heart were also isolated after 24 hours, 7 days and 14 days for Western analysis and comparison between periostin and TGFBI expression. The results show no expression in the sham-operated heart or 24 hours after MI injury, but a similar profile of induction at both 7 and 14 days after MI with periostin induction being more prominent (Fig 1C). Consistent with previous data, periostin localized to the peri-infarct area of hearts after 7days of MI and was deposited within the ECM region around the myocytes (Fig 1D) [12]. TGFBI was also localized to the infarct area of the heart 1 week after MI in a similar ECM-staining pattern (Fig 1D). Both gene products also showed substantial deposition around cardiac vessels (Fig 1D), especially after pressure overload stimulation as assayed by immunohistochemistry (S1 Fig).

### Cardiac phenotype of *Tgfbi* null mice with MI injury

**Fig 2:**
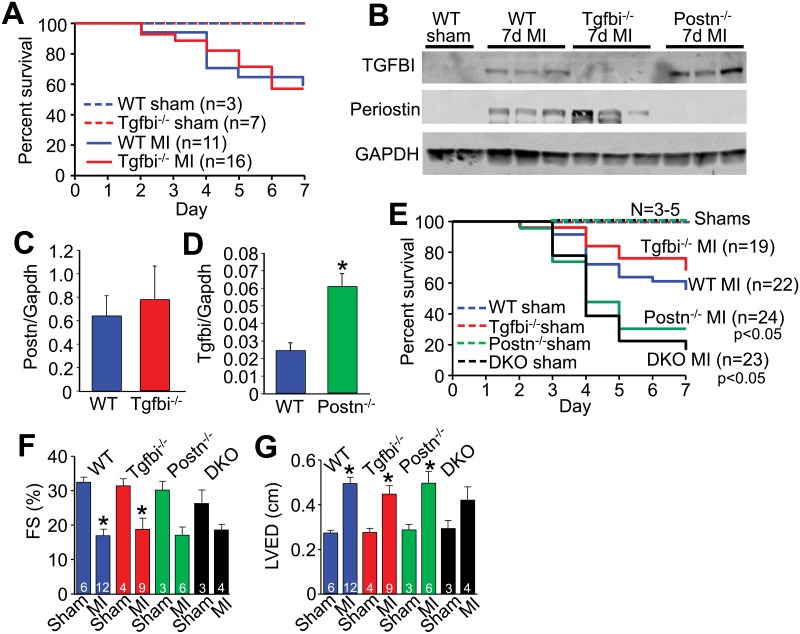
Loss of *Tgfbi* does not alter disease progression after MI. (A) Percent survival in the groups of mice shown over 7 days following MI surgery. No mortality was observed in sham-operated WT or *Tgfbi*^*-/-*^ mice. No differences in survival between WT and *Tgfbi*^*-/-*^ after MI was detected using the log rank Mantel-Cox statistical test. (B) Western blot analysis for TGFBI and periostin from isolated infarct areas of the hearts of the indicated groups of mice 7 days after MI. GAPDH was used as a loading control. (C and D) Quantification of protein levels normalized to GAPDH using densitometry for periostin in WT and *Tgfbi*^*-/-*^-operated hearts and for TGFBI in WT and *Postn*^*-/-*^-operated hearts. *p<0.05 vs WT using an unpaired Student’s T-test. (E) Percent survival in the indicated groups of mice over 7 days after sham or MI surgery. p<0.05 vs WT MI-operated animals using the log rank Mantel-Cox test (F) Percent ventricular fractional shortening (FS%) as measured by echocardiography in the indicated genotypes of mice 8 weeks after MI. *p<0.05 vs sham. (G) Left ventricular end diastolic dimension (LVED) as measured by echocardiography in the indicated groups of mice 8 weeks after MI. *p<0.05 vs sham using a parametric one way ANOVA with a Newman-Keuls post-hoc test.

Mice genetically engineered for global deletion of *Tgfbi* [18] were used to examine how induction of this protein following cardiac injury might functionally impact the heart. By comparison, *Postn*^*-/-*^ mice were previously shown to have greater lethality following MI injury due to inadequate scar formation and subsequent ventricular wall rupture [12, 13, 32]. However, here we observed that *Tgfbi*^*-/-*^ mice had no change in overall survival rates following MI injury compared with WT control mice (Fig 2A). The 30–40% of mice that died in each group following MI injury showed no difference in the rates of underlying ventricular wall rupture. To also investigate the possibility that periostin expression might compensate for the loss of TGFBI during the MI healing period we first analyzed protein levels within the MI region of the heart for both periostin and TGFBI. Indeed, we observed an increase in TGFBI protein levels in the hearts of *Postn*^*-/-*^ mice after MI compared with WT MI injured hearts, yet periostin protein levels where unchanged in the *Tgfbi*^*-/-*^ hearts after MI compared with WT mice with MI (Fig 2B, 2C and 2D). These results suggest that periostin is the more dominant family member expressed in the heart, as only in its absence is the paralog gene *Tgfbi* induced beyond what was observed in WT hearts after injury. Interestingly, in the lung where TGFBI has been reported to play a critical developmental role [18], bleomycin injury significantly induced TGFBI expression while periostin expression was decreased (S2 Fig), suggesting a reciprocal compensation pattern in this tissue compared with the heart (which also reflects the dominance of TGFBI in lung over periostin).

*Postn/Tgfbi* double null mice (DKO) were also generated and examined to further address compensation or the possibility that one gene dominates over the other in the heart following MI injury. Importantly, adult DKO mice displayed no baseline phenotype or other observable cardiac pathology up to 1 year of age (S3 Fig). As previously observed by us [12], *Postn*^*-/-*^ mice again showed greater lethality with higher rates of ventricular wall rupture following MI injury compared with WT controls (Fig 2E). However, separate groups of *Tgfbi*^*-/-*^ mice again showed no greater lethality than WT MI injured mice, while DKO mice showed a profile of lethality and ventricular wall rupture nearly identical to *Postn*^*-/-*^ mice, further suggesting that TGFBI protein induction is of little compensatory value during this injury response (Fig 2E). The same general reductions in cardiac ventricular fractional shortening or increases in left ventricular end diastolic dimension were detected between sham and MI operated groups of each genotype and were significantly changed by student T-test, but only some parameters were significant by ANOVA when all groups were compared together, and the later statistical results are shown in the figure as a conservative interpretation of the data (Fig 2F and 2G). However, the same trends were observed.

### Cardiac phenotype of *Tgfbi* null mice with TAC surgery

**Fig 3:**
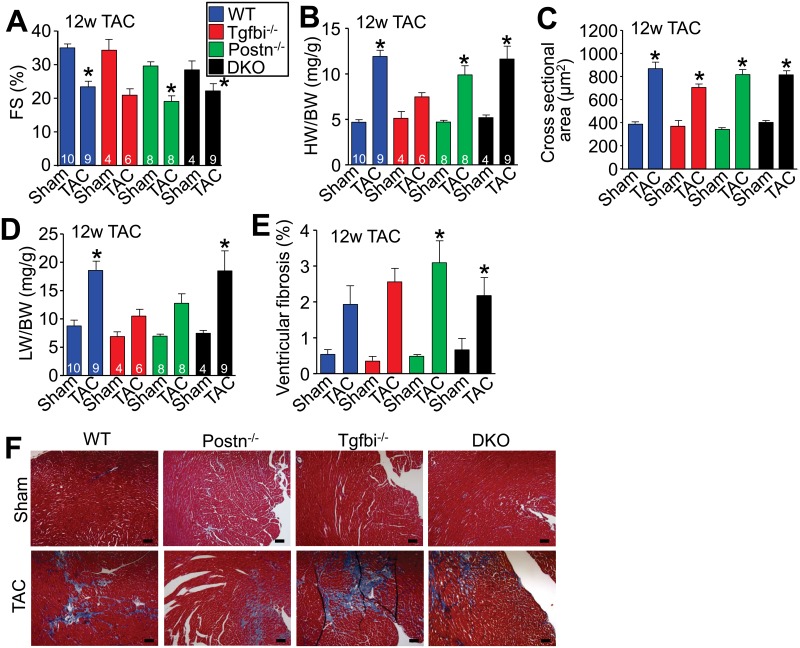
Loss of *Tgfbi* does not appreciably alter disease progression after long-term TAC. (A) Percent ventricular fractional shortening as measured by echocardiography 12 weeks after TAC in the indicated groups. *p<0.05 vs sham-operated controls. (B) Heart-weight normalized to body-weight in the indicated groups of mice after 12 weeks of TAC. *p<0.05 vs sham-operated controls. (C) Cardiomyocyte cross-sectional area from ventricular histological sections in the indicated groups of mice 12 weeks after MI. *p<0.05 vs sham-operated controls. Four to six hearts were processed from each group, with at least 3 fields analyzed per histological section. (D) Lung-weight normalized to body-weight in the indicated groups of mice 12 weeks after TAC. *p<0.05 vs sham-operated controls. (E) Ventricular fibrosis from Masson's trichrome-stained histological images in the indicated genotypes of mice after 12 weeks of TAC. *p<0.05 vs sham-operated controls. Four to six hearts were processed from each group, with at least 3 fields analyzed per histological section. (F) Representative Masson’s trichrome-stained histological heart sections from sham and TAC hearts after 12 weeks of MI. Images were taken at 100x magnification. Scale bar = 100 μm. All statistical analyses were performed using a parametric one way ANOVA with a Newman-Keuls post-hoc test.

To further analyze the role of TGFBI during cardiac injury, mice were subjected to long-term pressure overload stimulation by TAC, which induces a fibrotic response and reduces cardiac functional performance. After 12 weeks of TAC all genotypes of mice showed reductions in ventricular fractional shortening (Fig 3A), increases in heart-weight normalized to body-weight (Fig 3B), increases in the cross-sectional area of cardiomyocytes in the left ventricle (Fig 3C) and a roughly similar increase in total ventricular fibrosis (Fig 3E and 3F). Interpretation of statistical significance behind these results was based on ANOVA, although comparison by student T-test between the WT group and any specific genotype showed a significant difference. However, *Postn*^*-/-*^ and *Tgfbi*^*-/-*^ mice even by ANOVA showed less pulmonary congestion after 12 weeks of TAC compared with WT controls or the DKO mice (Fig 3D). Pulmonary congestion is often used as an indicator of heart failure. We also observed that *Tgfbi*^*-/-*^ mice displayed a decrease in hypertrophy following 12 weeks of TAC compared with all the other genotypes of mice by any statistical test (Fig 3B), although we are uncertain as to why this might be the case. Importantly, there were no differences between the genotypes in pressure gradients across the aortic constrictions. In summary, the data suggest that loss of *Tgfbi* or the loss of both *Tgfbi* together with *Postn* (DKO mice) does not appreciably worsen the cardiac phenotype of mice following pressure overload stimulation.

### Heart specific over-expression of TGFBI

**Fig 4:**
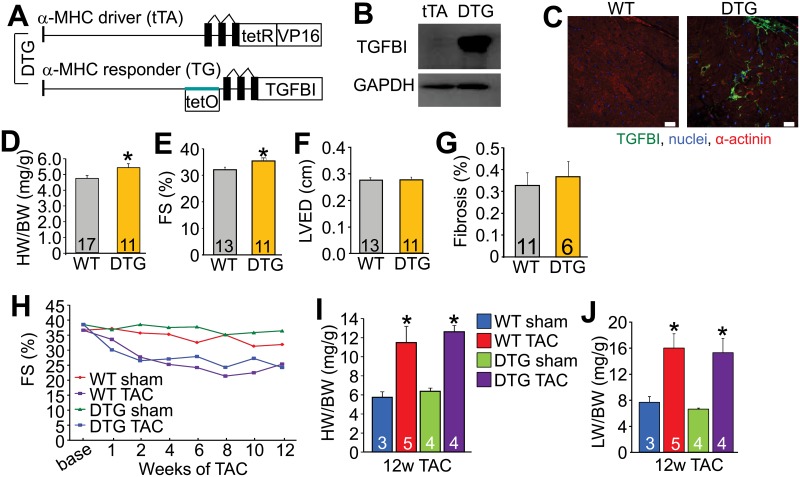
Cardiac-specific over-expression of TGFBI. (A) Schematic representation of the double transgenic (DTG) system used to generate cardiac-specific, doxycycline inducible expression of TGFBI. (B) Western blot analysis of TGFBI in hearts of DTG mice. GAPDH was used as a control. (C) Immunohistochemistry of heart histological sections at 6 months of age from WT or TGFBI DTG mice. Sections were imaged for the proteins or markers shown. Scale = 10 μm. (D) Heart-weight (HW) normalized to body-weight (BW) in WT and DTG mice at 6 months of age. *p<0.05 vs WT using an unpaired Student’s T-test. (E) Percent fractional shortening (FS%) in WT and DTG mice at 6 months of age. *p<0.05 vs WT using an unpaired Student’s T-test. (F) Left ventricular dimension in diastole (LVED) in WT and DTG mice at 6 months of age as measured by echocardiography. (G) Percent fibrosis measured from Masson’s trichrome-stained cardiac histological sections from WT and DTG mice at 1 year of age. (H) Assessment of FS% in the indicated groups of mice over 12 weeks after sham or TAC surgery. (I) HW normalized to BW in the indicated groups of mice after 12 weeks after TAC. *p<0.05 vs corresponding sham group. (J) Assessment of lung-weight (LW) normalized to BW in the indicated groups of mice after 12 weeks of TAC. *p<0.05 vs corresponding sham group analyzed by parametric one way ANOVA with a Newman-Keuls post-hoc test.

We previously showed that periostin over-expression in the heart using a transgene approach resulted in a mild but significant degree of cardiac hypertrophy by 32 weeks of age compared with WT controls, although over-expression of this protein was otherwise innocuous to the heart and did not increase fibrosis [12]. Here we generated transgenic mice with inducible over-expression of TGFBI under control of the α-MHC binary tetracycline transactivator (tTA) regulated system to determine how this protein might impact the heart (Fig 4A) [22]. Western analysis showed robust over-expression of TGFBI in the heart in the double transgenic mice (DTG) (Fig 4B). Immunohistochemical analysis of hearts from these DTG mice showed accumulation of secreted TGFBI protein within the ECM between the cardiomyocytes of the adult heart similar to its deposition when normally secreted by stress-activated fibroblasts (Fig 4C). TGFBI DTG mice also displayed a mild phenotype of concentric cardiac hypertrophy with aging (Fig 4D) that was also associated with an increase in ventricular fractional shortening (Fig 4E), but without a change in left ventricular end diastolic dimension or fibrosis (Fig 4F and 4G). Following long-term TAC, TGFBI DTG mice showed the same decrease in ventricular fractional shortening, increases in heart-weight normalized to body-weight, and increases in lung-weight normalized to body-weight as WT controls with TAC (Fig 4H, 4I and 4J). Together these data suggest that over-expression of TGFBI acts similar to previous results with periostin over-expression [12], in that it appears to support a greater cardiac growth response at baseline without impacting fibrosis or the ability of these hearts to compensate to long-term pressure overload.

## Discussion

Periostin is a matricellular protein that is induced and secreted specifically by activated fibroblasts in areas of tissue injury where it accumulates within the ECM to facilitate type 1 collagen maturation and crosslinking, as well as strengthening of the ECM by interactions with tenascin-C, fibronectin and collagen V [33–35]. TGFBI and periostin are family members that contain 4 fasciclin domains with conserved sequence similarity. Similar to periostin, TGFBI is also induced by transforming growth factor-β (TGFβ) signaling in areas of tissue injury and is secreted by activated fibroblasts [4, 36]. While both *Postn* and *Tgfbi* gene expression are induced by injury events in nearly all tissues evaluated, *Postn* induction has been most heavily evaluated in the heart following injury or in cancer cells as they undergo invasion with ECM remodeling [12] [37–39]. Previous work from us and others has shown that periostin is required for proper scar formation in the heart after MI injury, such that mice deficient in this gene undergo lethal rupture [12, 13]. This event was shown to be specific to the cardiac fibroblast as targeted ablation of periostin-expressing fibroblasts prevented adverse remodeling in mice after MI injury or angiotensin II infusion [32]. We also showed that deletion of periostin-expressing activated fibroblasts from the heart during MI injury compromised scar formation and results in lethal ventricular wall rupture [40].

To date, the majority of the literature pertaining to TGFBI has focused on corneal dystrophy. TGFBI is a primary disease-causing gene associated with corneal dystrophy where mutations cause protein deposits on the cornea eventually leading to blindness [15, 16]. *Tgfbi* null mice were also recently shown to have a pathological lung phenotype as discussed earlier [18]. While a single description of *Tgfbi* mRNA induction in the diseased heart from an extensive array profiling study of the mouse heart was reported [21], almost nothing is known of TGFBI function in the heart. Moreover, nothing was previously known of the potential for periostin and TGFBI to function redundantly or if each might possibly have unique roles in selective tissues based on where they are primarily expressed.

Here we observed that TGFBI is induced in the heart with injury, and that this expression is within fibroblasts. However, TGFBI expression and ECM deposition was noticeably less compared with periostin upon cardiac injury, as assessed by Western blotting and immunohistochemistry. However, lung injury showed the opposite effect with a much greater induction of TGFBI compared to periostin, hence it is likely that differences in phenotypes and disease profiles between *Postn* and *Tgfbi* gene-deleted mice is a reflection of where each gene is primarily expressed across selective tissues. Indeed, this hypothesis is supported by previous work where periostin and TGFBI are reported to have opposite effects in the lung in mouse models of bronchopulmonary dysplasia [18, 41]. In response to hyperoxia, *Postn* null mice displayed normal alveolar development [41] while *Tgfbi* null mice at baseline showed alveolar defects [18].

In the heart, mice deficient for *Postn* attempted at compensate after MI injury by upregulation of *Tgfbi* expression. However, loss of the less highly expressed *Tgfbi* gene in the heart during MI injury did not result in greater induction of *Postn* expression, as periostin induction is presumably already more than sufficient. In support of this conclusion, *Postn/Tgfbi* double null mice showed the same degree of defective scar formation and ventricular wall rupture after MI as *Postn* single null mice, while single null *Tgfbi* mice were unaffected relative to WT.

To further address if TGFBI and periostin might function similarly in the mouse heart, apart from differences in expression levels, we engineered TGFBI transgenic mice with heart-specific expression. TGFBI over-expression, which was deposited within the ECM, induced a mild degree of concentric cardiac hypertrophy reminiscent of a nearly identical increase of baseline cardiac hypertrophy observed in periostin heart-specific over-expressing mice at 32 weeks of age [12]. Neither periostin nor TGFBI over-expression was sufficient to induce a fibrotic response or otherwise impact pressure overload induced fibrosis. These results suggest that periostin and TGFBI have similar function at the protein level; further suggesting that differences in the disease susceptibility phenotype observed between mice lacking either gene is a reflection of expression dominance in the heart. In the future it would be interesting to insert the TGFBI cDNA into to *Postn* locus and create homozygous targeted mice, which if our hypothesis is correct, such mice lacking periostin protein would be fully compensated by TGFBI protein induction from the *Postn* locus because it drives much higher expression in cardiac fibroblasts with injury events.

Finally, while periostin and TGFBI are highly similar through their 4 fasciclin domains [42], periostin uniquely contains a C-terminal region that is alternatively spliced [42–44], which when cleaved can presumably contribute to alterations in protein function [14, 34, 38]. In fact, it has been shown that periostin binding to tenascin-C, fibronectin and collagen V is enhanced upon loss of the C-terminal tail [33, 35]. These data suggest that the dynamic C-terminal tail of periostin, which is lacking in TGFBI, may give this family member unique functional characteristics. More studies are clearly needed to further dissect the functional similarities and differences between these two related family members in the heart and other tissues.

## Supporting information

S1 Fig TGFBI is induced in TAC hearts. Immunohistochemical analysis of sham and TAC-operated hearts 12 weeks after surgery stained for TGFBI in green (A) or periostin in green (B). Red is α-actinin. Blue stain is DAPI for nuclei. Images were taken at 200x magnification and the scale bar = 50 μm.
(PDF)

S2 Fig Periostin and TGFBI are differentially regulated in a model of lung injury in the mouse. (A) Western blot analysis of TGFBI and periostin expression in lung tissue treated intranasally for 3 weeks with either bleomycin or saline. GAPDH served as a loading control. (B) Quantification of TGFBI protein expression normalized to GAPDH in saline and bleomycin treated lungs. *p<0.05 vs saline. (C) Quantification of periostin protein expression normalized to GAPDH in saline and bleomycin treated lungs. *p<0.05 vs saline. An unpaired students T-test was used for statistical analysis in B and C.
(PDF)

S3 Fig Baseline characterization of WT, *Tgfbi*^*-/-*^, *Postn*^*-/-*^, and DKO mice. (A, C) Ventricle-weight normalized to body-weight (VW/BW) of the indicated genotypes of mice after 6 months (A) and 1 year (C) of aging. (B, D) Percent ventricular fractional shortening (FS%) as measured by echocardiography after 6 months (B) and 1 year (D) of aging. Statistical analysis was performed using a parametric one way ANOVA with a Newman-Keuls post-hoc test. (E) Representative Masson’s trichrome-stained cardiac histological sections shown at 100x magnification (6 months of age). Scale bar = 100 μm.
(PDF)
