# Supplementary figures and images for: TGFBI functions similar to periostin but is uniquely dispensable during cardiac injury
Source: PLoS One. 2017 Jul 27;12(7):e0181945. doi: 10.1371/journal.pone.0181945 (PMC5531541; doi:10.1371/journal.pone.0181945)

**A**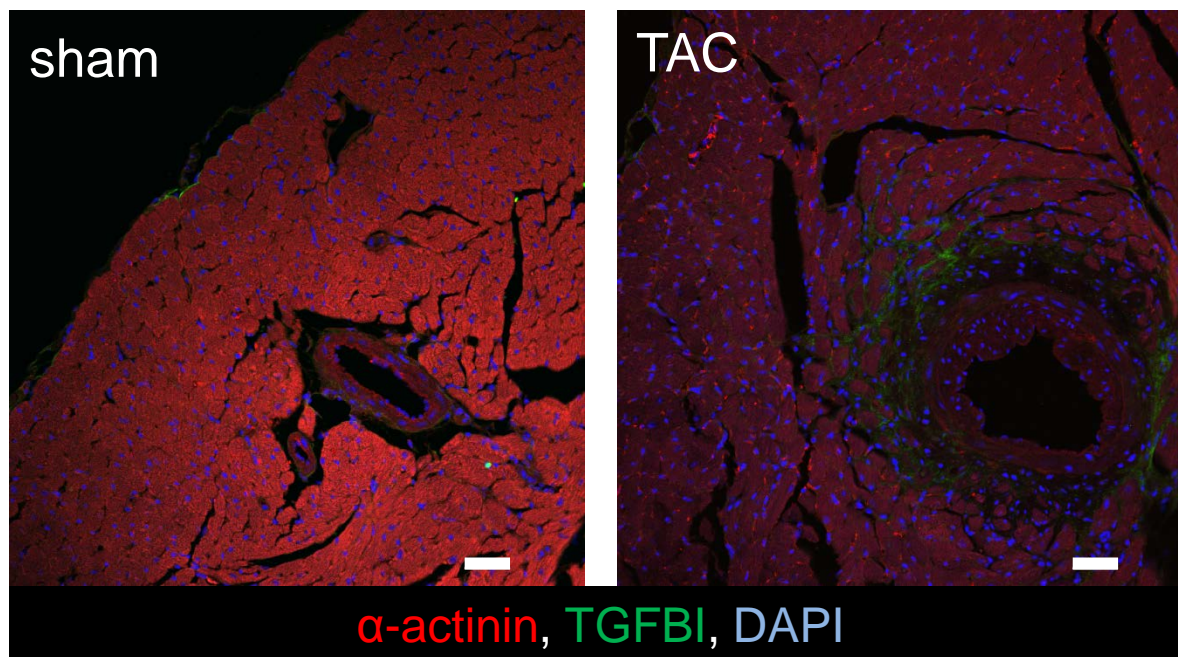**B**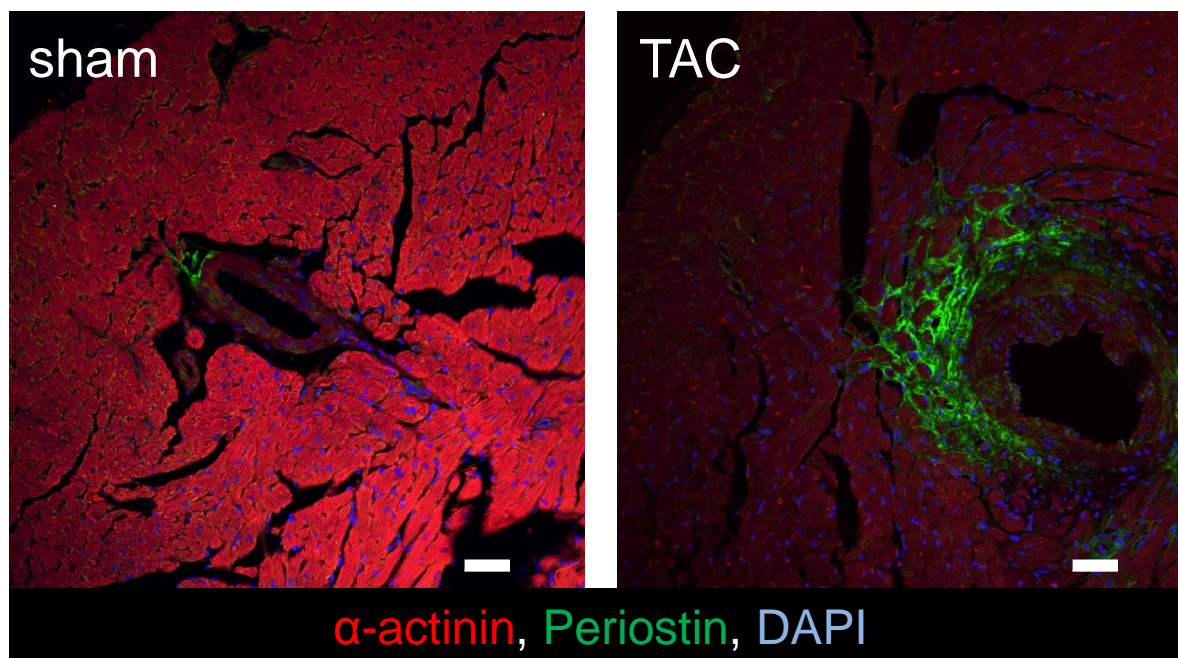

Supplement: S1 Fig — Immunohistochemical analysis of sham and TAC-operated hearts 12 weeks after surgery stained for TGFBI in green (A) or periostin in green (B). Red is α-actinin. Blue stain is DAPI for nuclei. Images were taken at 200x magnification and the scale bar = 50 μm. (PDF) [file pone.0181945.s001.pdf]

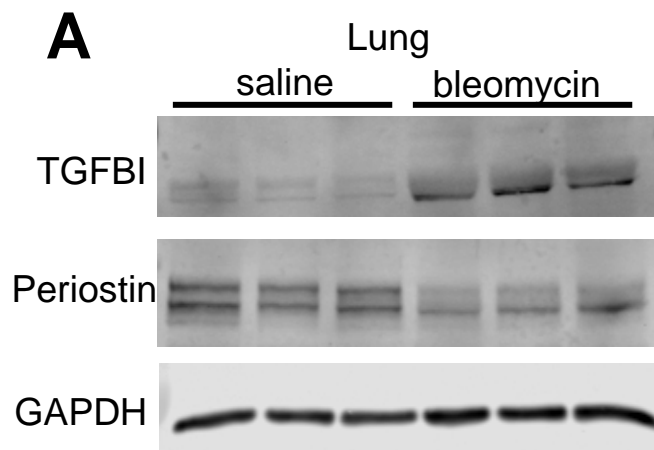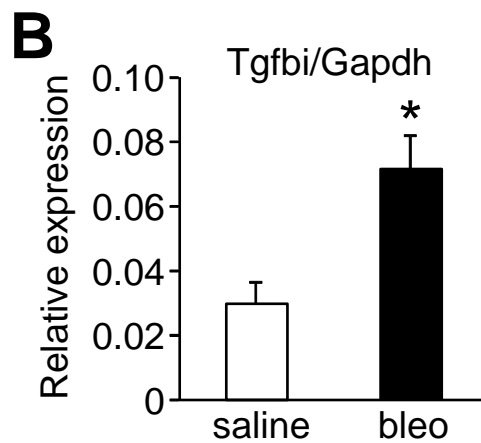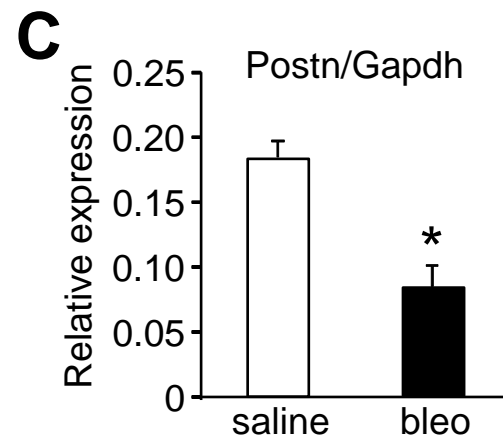

Supplement: S2 Fig — (A) Western blot analysis of TGFBI and periostin expression in lung tissue treated intranasally for 3 weeks with either bleomycin or saline. GAPDH served as a loading control. (B) Quantification of TGFBI protein expression normalized to GAPDH in saline and bleomycin treated lungs. *p<0.05 vs saline. (C) Quantification of periostin protein expression normalized to GAPDH in saline and bleomycin treated lungs. *p<0.05 vs saline. An unpaired students T-test was used for statistical analysis in B and C. (PDF) [file pone.0181945.s002.pdf]

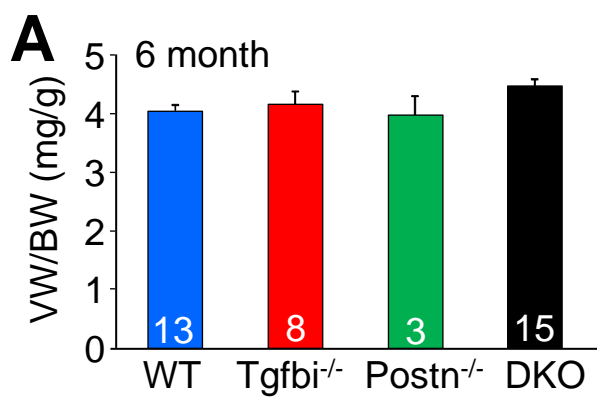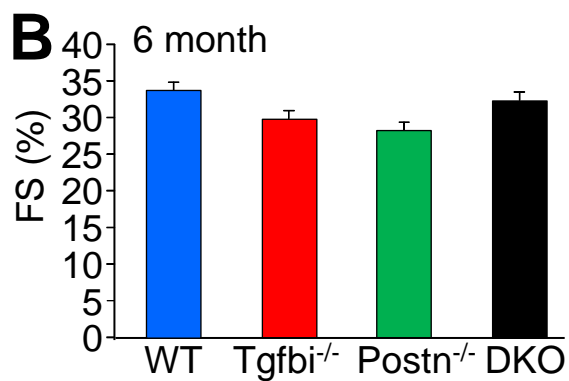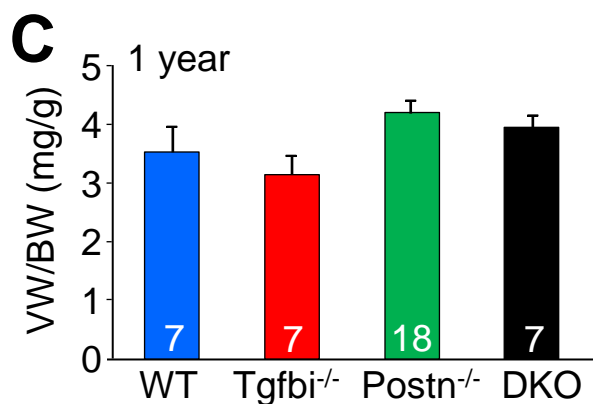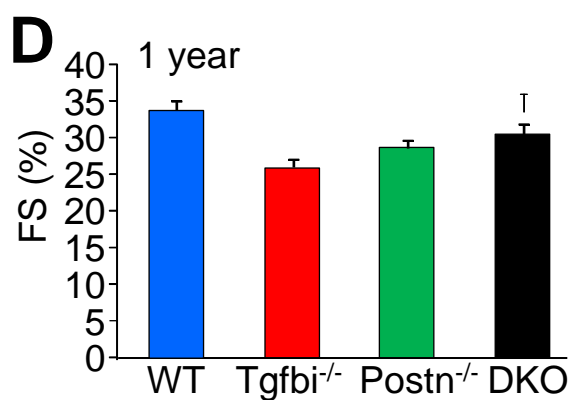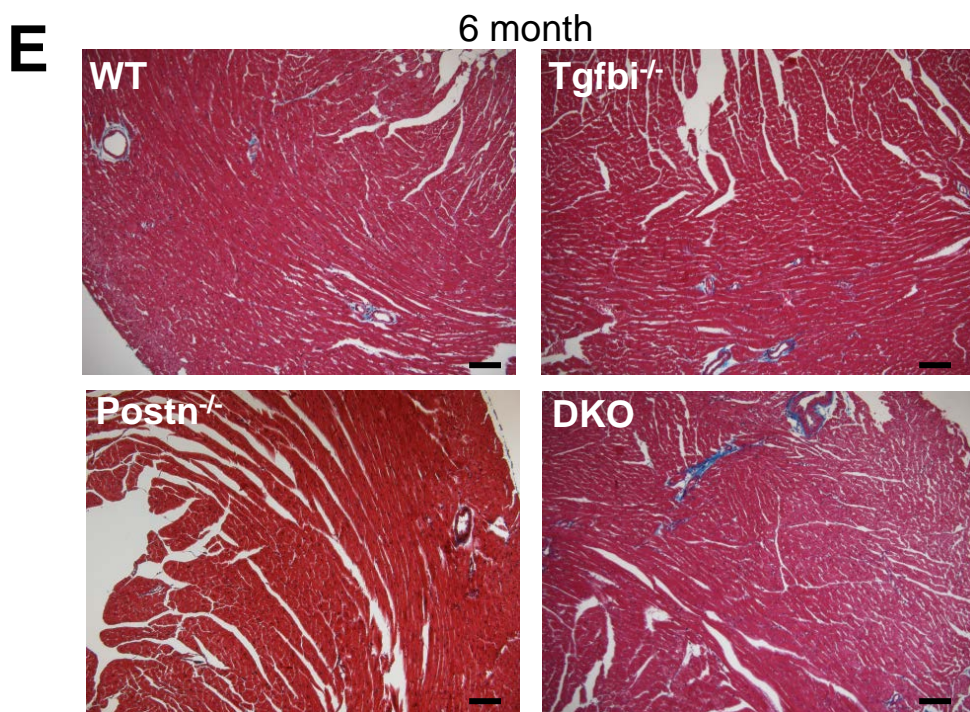

Supplement: S3 Fig — (A, C) Ventricle-weight normalized to body-weight (VW/BW) of the indicated genotypes of mice after 6 months (A) and 1 year (C) of aging. (B, D) Percent ventricular fractional shortening (FS%) as measured by echocardiography after 6 months (B) and 1 year (D) of aging. Statistical analysis was performed using a parametric one way ANOVA with a Newman-Keuls post-hoc test. (E) Representative Masson’s trichrome-stained cardiac histological sections shown at 100x magnification (6 months of age). Scale bar = 100 μm. (PDF) [file pone.0181945.s003.pdf]
